# Thinking beyond the colon-small bowel Involvement in clostridium difficile infection

**DOI:** 10.1186/1757-4749-1-7

**Published:** 2009-03-19

**Authors:** Udayakumar Navaneethan, Ralph A Giannella

**Affiliations:** 1 Division of Digestive Diseases, Department of Internal Medicine, University of Cincinnati College of Medicine, Cincinnati, Ohio, USA

## Abstract

Small intestinal *Clostridium difficile *seems to be increasing in incidence. The spectrum of *Clostridium difficile *infection (CDI) has definitely expanded with small bowel involvement. They are more frequently reported in patients with inflammatory bowel disease (IBD) who have undergone total colectomy or patients with Ileal anal pouch anastomosis. The most common presentation is increased ileostomy output with associated dehydration. High clinical suspicion, early recognition and appropriate treatment are the keys to successful resolution. The increase in the number of these patients may actually reflect an increase in the rising incidence of CDI in general or increasing virulence of the organism. Heightened public awareness and initiation of prompt preventive measures are the keystones to control of this infection. This disease is no longer limited to the colon and physicians should be educated to think beyond the colon in patients with CDI.

## Review

*Clostridium difficile *infection (CDI) is traditionally thought off, as a disease of the colon frequently with pseudomembrane formation. We have recently encountered three cases of CDI involving the small intestine; a clinical situation which we believe is poorly appreciated. The following brief case report highlights an increasingly common clinical scenario.

A 38-year-old woman with history of Crohn's disease (CD) presented with nausea, vomiting, and abdominal pain. Her past medical history was significant for CD with colonic and small bowel involvement with history of total colectomy with end ileostomy 4 years prior for medically refractory disease. The patient also reported that she had been changing her ileostomy bag every 6 h for 4–5 days prior to presentation. Her labs were significant for acute renal failure with a blood urea nitrogen and serum creatinine of 56 and 2.94 at admission and leukocytosis. Stool studies were sent from the ileostomy fluid and the studies reported positive for *Cl. difficile *toxin A and B by enzyme immunoassay. Patient was diagnosed with small bowel CDI and was started on oral vancomycin (125 mg q.i.d.), which resulted in resolution of the symptoms (within 48 h) and decrease in the ileostomy output and her repeat stool studies for CDI became negative. Patient subsequently completed 10 days of vancomycin treatment and has been asymptomatic in the ensuing two months.

This clinical situation epitomizes the expanding clinical spectrum of CDI which in spite of several recent reports of small bowel involvement is poorly appreciated. Although colonization of the small bowel by *Cl. difficile *was reported almost three decades before and the organism was isolated from the jejunal segments in autopsy and from the jejunal aspirate of patients with chronic diarrhea [1,2], reports of small bowel CDI were rare until recently. Thus the small intestine may serve as a reservoir and certain factors could precipitate disease and infection. 34 patients with *Cl. difficile *enteritis are reported thus far in the literature. [3-20] It is surprising that only 14 case reports were reported from 1980 to 2006 and the remaining reported over the past two years. With the expanding spectrum of CDI, physicians need to be aware and suspect and treat this infection.

## Pathogenesis

The risk of CDI involving the colon increases with antibiotic use, intensive care unit or prolonged hospital stay, increasing age, a defective immune system and contact with infected patients. Studies from nationwide inpatient databases also demonstrate an increase in incidence and severity of CDI of the colon in patients with inflammatory bowel disease (IBD) [21-23].

In patients with small bowel CDI, the risk factors seem to be slightly different. Antibiotic use and IBD predispose to small bowel CDI similar to CDI of the colon. 31/34 patients received antibiotics before the development of small bowel CDI [3-20]. Prior surgeries of the colon/colectomy, and host factors including advanced age, immunocompromised state are proposed as additional risk factors for small bowel CDI [16]. More than 90% of patients (31/34) reported in the literature had gastrointestinal surgery of the colon.

The reason for the predisposition of patients who undergo colonic surgery to small bowel CDI is not clear although multiple hypotheses can be offered. Firstly, after colectomy changes occur in the small-bowel bacterial flora such that the neoterminal ileum is colonized by colonic-type bacterial flora [24], which may make it susceptible to overgrowth with *Cl. difficile*, particularly with concomitant antibiotic use [10]. Similarly, changes occur in the ileostomy flora which comes to resemble the fecal flora [25]. The fecal stream also changes the mucosa of the small bowel to undergo metaplastic changes. This occurs in patients with Ileal anal pouch anastomosis (IPAA) in which the epithelium of pelvic ileoanal pouches undergo morphologic changes including mild villus atrophy, paneth cell hyperplasia, and a partial transition to colonic mucin phenotype without complete metaplasia, all of which facilitate fecal flora establishment [26,27]. Similar changes may occur in patients with end ileostomy and the long latent period between the surgery and CDI supports this as 11 of the 31 patients had surgeries performed between 1 and 52 years before developing enteritis [3-20]. In patients who develop infection in the immediate post operative period, many had CDI of the colon prior to surgery which suggests the hypothesis that in those patients CDI of the small bowel may be secondary to migration of the *Cl. difficile *into the small bowel after surgery.

The pathogenesis of CDI of the colon is toxin mediated through colonic receptors for *Cl. difficile *toxin. The nature of the interaction of *Cl. difficile *toxin(s) with the small bowel epithelium is unclear although the sucrase-isomaltase complex, a small intestinal brush border enzyme, has been proposed to contain a receptor for *Cl. difficile *toxin (s) [28]. Toxin interaction with this glycoprotein complex could mediate diarrhea in the immediate post operative period [29]. Secondly, colonization of the small bowel occurs because the protective mechanisms are compromised by colonic resection surgeries [8]. The mechanical action of the ileocecal valve may be lost because of surgery. Surgeries involving only the left side of the colon with preservation of the ileocecal valve do not seem to increase the risk of CDI of the small bowel highlighting the importance of the ileocecal valve in preventing colonization.

## Clinical presentation

Increasing ileostomy output seems to be the most common clinical presentation of small bowel CDI. Increased ileostomy output was the presenting manifestation in 16/17 patients in two large case series [16,17]. Increased ileostomy output is severe enough in some patients to result in acute renal failure and in fact in the largest series to date, 8/11 presented with acute renal failure [17].

Also it is interesting to note that more than 50% of patients (19/34) with small bowel CDI had IBD. 18 patients had end ileostomy and one had IPAA following ulcerative colitis (UC). 13 of these patients with ileostomies had UC and the other five patients had CD. The rising incidence of CDI should raise suspicion of *Cl. difficile *when IBD patients develop profuse diarrhea, particularly in patients who underwent recent surgical intervention. The susceptibility of IBD patients to CDI both of the colon and small bowel is intriguing. Although IBD patients are at risk because of antibiotic use, hospitalizations, the dramatic risk of infection cannot be fully explained by the above risk factors and raises the question whether abnormalities in mucosal immune response in IBD might play a role in CDI.

## Treatment and prognosis

Initial reports highlighted that infection of the small bowel with *Cl. difficile *was associated with increased mortality [3-15]. The increased permeability of the small intestinal mucosa was hypothesized to be due to result in profound sepsis [8]. Also the presence of several comorbidities could have increased the risk of complications associated with infection. However recent reports are associated with good prognosis; in fact, two large recent case series reported no mortality [16,17]. This may be secondary to earlier diagnosis and detection of the problem and early initiation of treatment.

The optimal treatment of small bowel CDI is unclear and stratification of the disease severity as CDI of the colon could be used to initiate an appropriate management plan. In a series of 11 patients, more than 50% responded to metronidazole alone [17]. In another series, all the six patients were treated with a combination of metronidazole and vancomycin [16]. Thus similar to colonic CDI, vancomycin may be the first line treatment for severe CDI, while in mild to moderate disease, metronidazole may be used; however switching to vancomycin in patients who do not improve within 72 hours of initiation of treatment with metronidazole would seem appropriate.

## Conclusion

Small intestinal *Cl. difficile *seems to be increasing in incidence. The spectrum of CDI has definitely expanded with small bowel involvement. They are more frequently reported in patients with IBD who have undergone total colectomy or patients with IPAA. The most common presentation is increased ileostomy output with associated dehydration. High clinical suspicion, early recognition and appropriate treatment are the keys to successful resolution. The increase in the number of these patients may actually reflect an increase in the rising incidence of CDI in general or increasing virulence of the organism. Heightened public awareness and initiation of prompt preventive measures are the keystones to control of this infection. This disease is no longer limited to the colon and physicians should be educated to think beyond the colon in patients with CDI.

## Abbreviations

CD: Crohn's disease; CDI: *Clostridium difficile *infection; IBD: Inflammatory bowel disease; IPAA: Ileal anal pouch anastomosis; UC: Ulcerative colitis.

## Competing interests

The authors declare that they have no competing interests.

## Authors' contributions

UN drafted the manuscript. RG conceived of the topic, and participated in manuscript editing and revisions. All authors read and approved the final manuscript.
